# Cost and cost-effectiveness of pediatric home-based versus facility-based TB Preventive Treatment in Ethiopia (CHIP-TB)

**DOI:** 10.1371/journal.pgph.0004466

**Published:** 2025-04-30

**Authors:** Akash Malhotra, Ahmed Bedru, Fiseha Mulatu, Bareng A.S. Nonyane, Silvia Cohn, Christiaan Mulder, Samuel Bayu, Stephanie Borsboom, Gidea Conradie, Jonathan E. Golub, Richard E. Chaisson, Gavin Churchyard, David W. Dowdy, Hojoon Sohn, Nicole Salazar-Austin

**Affiliations:** 1 Department of Epidemiology, Johns Hopkins Bloomberg School of Public Health, Baltimore, Maryland, United States of America; 2 Department of Global Health, University of Washington, Seattle, Washington, United States of America; 3 K.N.C.V. Ethiopia, Addis Ababa, Ethiopia; 4 Department of International Health, Johns Hopkins Bloomberg School of Public Health, Baltimore, Maryland, United States of America; 5 Department of Medicine, Johns Hopkins School of Medicine, Baltimore, Maryland, United States of America; 6 KNCV Tuberculosis Foundation, The Hague, The Netherlands; 7 Amsterdam Institute for Global Health and Development, Amsterdam University Medical Centres, Amsterdam, The Netherlands; 8 The Aurum Institute, Johannesburg, South Africa; 9 School of Public Health, University of Witwatersrand, Johannesburg, South Africa; 10 Department of Medicine, Vanderbilt University, Nashville, Tennessee, United States of America; 11 Department of Preventive Medicine, Seoul National University College of Medicine, Seoul, South Korea; 12 Department of Human Systems Medicine, Seoul National University College of Medicine, Seoul, South Korea; 13 Institute of Health Policy and Management, Seoul National University College of Medicine, Seoul, South Korea; 14 Department of Pediatrics, Johns Hopkins School of Medicine, Baltimore, Maryland, United States of America; Federal University of Rio de Janeiro, BRAZIL

## Abstract

Tuberculosis preventive treatment (TPT) is an essential intervention recommended for all child contacts in Ethiopia under 15 years who are at risk of tuberculosis (TB) infection. We conducted a cost and cost-effectiveness analysis of home-based versus facility-based TPT provision for child contacts in Ethiopia. As part of the CHIP TB trial, a pragmatic, cluster-randomized trial conducted at eighteen clinics in Ethiopia, clinics were randomized to either a home-based model (intervention arm), administered by community health workers, or a facility-based model (standard of care) for managing child contacts. Cost data were collected from both a health service perspective and a household perspective, capturing all costs relevant to TPT. Costs were evaluated on a per-household basis, with the primary outcome being the difference in median costs per household initiating TPT. A secondary outcome assessed the cost-effectiveness as the incremental cost per additional child contact starting TPT. Probabilistic sensitivity analyses (PSA) were conducted to examine the robustness of findings. At an average cost of US$18.92 per household managed, Home-based contact management, including TPT delivery was cost-saving compared to facility-based TPT delivery (US$27.24 per household managed) assessed based on the partial-societal perspectives, largely due to reductions in household out-of-pocket costs. The home-based strategy was both less costly and had increased TPT initiation in 61.5% of the scenarios assessed in the PSA. Home-based contact management is a cost-saving alternative for households and provides comparable initiation rates to facility-based care, making it a feasible approach to improve TB preventive treatment accessibility. Although it does not entirely replace facility-based care, a hybrid model that respects household preferences and allows flexibility in delivery could enhance TB care access for socio-economically disadvantaged households, potentially reducing health inequities. The trial was registered on clinicaltrials.gov NCT04369326 on April 30, 2020. https://clinicaltrials.gov/study/NCT04369326.

## Introduction

In 2023 there were around 1.25 million children aged <15 years suffering from tuberculosis (TB) disease, accounting for 12% of the global burden [1]. Of these, about 200,000 died, amounting to a case fatality ratio of 16% [2]. The first United Nations High-Level Meeting (UNHLM) for TB in 2018 set a target to alleviate this burden in children and aimed to provide tuberculosis preventive treatment (TPT) to 4 million child household contacts under 5 years and 20 million household contacts over 5 years from 2018-2022 [3]. Although short course TPT is both an effective and cost-effective intervention for child household contacts [4], only 2.2 million (55% of the UNHLM target) of the child contacts received TPT [3]. This shortfall and significant TB disease burden faced by children underscores the urgent need for innovative and household-centered implementation of TPT for children in high TB-burden, resource limited settings.

In high-burden, low-resource settings, the current standard of care for TPT delivery to children—namely TB contact investigation and TPT initiation at health facilities—is plagued with challenges such as long wait times, scheduling, and high workload [5]. Ethiopia is one such setting with an estimated 188,000 new TB cases and 29,000 TB-related deaths in 2023 making it one of the 30 high burden TB countries [6,7]. The costs and time to travel to a health facility act as deterrents for most households, disproportionately impacting those who are socio-economically disadvantaged [8,9]. Household contact investigation for people newly diagnosed with TB, as recommended by the World Health Organization (WHO), offers an opportunity to treat eligible children with TPT [10]. TPT provision at home, can both help meet UNHLM 2023 TPT targets and offset many challenges linked to seeking care at the health facility. Via task-sharing, community health workers (CHWs) in Ethiopia, who provide a package of 18 interventions through outreach and home visits, can play a pivotal role in TPT initiation and follow up for eligible children at the household level, reducing the burden on more experienced health professionals at the facility [11].

The findings of the CHIP-TB trial, a pragmatic, cluster-randomized trial conducted at eighteen clinics in Ethiopia randomized to either home-based (intervention) or facility-based (standard of care) child contact management, indicated that home-based contact management was acceptable, feasible, and more household-centered compared to facility-based care. TPT initiation and completion rates were similar between the two groups, suggesting that home-based contact management is as effective as facility-based care. For every TB client, the home-based arm initiated more children on TPT compared to the facility-based arm, 1.7 vs 1.3, though this difference was not statistically significant (p value = 0.3). Additionally, the home-based arm identified more child contacts, below age 15, per TB client, as in the facility-based arm, 1.9 vs 1.5, and this difference was also not statistically significant (p value = 0.3). Although provision of TPT is likely cost-effective in this population [4], empiric evidence is lacking on the resource implications of a more household-centered approach such as home-based TPT delivery and contact management. An intervention that provides TPT care at home will likely be more resource intensive to the health system than one provided at the clinic. However, when considering costs to households—encompassing both opportunity costs and direct expenses related to clinic visits—some of these costs may be offset.

From the perspective of a household with a child contact for TB, we hypothesized home-based contact management delivered by CHWs to reduce out-of-pocket and indirect costs for the household, compared to the facility-based standard of care. From the health system perspective, the travel cost and time devoted away from the health facility, potentially makes the intervention more expensive than the standard of care.

## Methods

### Ethics statement

The study protocol was submitted and approved by the WHO Ethics Research Committee (ERC.0003241), the Oromia Regional Health Bureau Research Ethics Review Board (KNCV/7/15/21), and the Office of Human Subject Research Institutional Review Boards of Johns Hopkins (IRB00249787).

### Study design and participants

CHIP-TB was a cluster-randomized trial conducted at thirty-six clinics with decentralized TB services (eighteen each in Ethiopia and South Africa) [12]. The costing study was nested within the CHIP-TB trial. Participants for the costing study in Ethiopia, the focus geography of this analysis, interacted with the costing study team from February 7, 2022 to August 30, 2022. Participation in the costing study was voluntary. Clinics (clusters) were randomized 1:1 to either home-based (intervention by task sharing of TPT care by CHWs) or facility-based (standard of care by staff at health facilities) contact management [12]. The trial eligibility criteria was pragmatic and included all TB clients and their child contacts who were eligible for TPT [12]. The primary aim of the trial in Ethiopia was to assess the cluster-level ratio of the number of close child contacts <15 years old initiated on TPT per TB client [12]. Following Ethiopia’s TB guidelines, TPT could mean 3 months of weekly rifapentine and isoniazid (3HP) for children ≥ 2 years, three months of daily rifampicin and isoniazid (3RH) for children < 2 years of age and as an alternate for children ≥ 2 years, and six months of daily isoniazid (6H) for children with drug-drug interactions to rifamycins [12].The protocol, which includes the costing analysis plan, has been published previously [12].

### Inclusivity in global research

Additional information regarding the ethical, cultural, and scientific considerations specific to inclusivity in global research is included in the supporting information files (S1 Checklist).

### Informed consent

Written consent from the TB client and the caregivers (who may or may not have been the TB client) was sought to collect their child’s data and for TPT services in the home [12]. Separate consent was sought from the caregiver for participation in two costing interviews [12]. Healthcare workers and caregivers were asked to provide consent for time and motion observations [12]. Assent was obtained for all child contacts 12 years and older [12].

### Procedures

We estimated the cost and cost-effectiveness of the intervention using a health system and household perspective. Likewise, cost data were collected and assessed for both the health service delivery as well as those borne by the study participant household. Trained research assistants (RAs) were each assigned to intervention and standard of care clinics to collect cost data.

Across the eighteen clinics, we collected participant perspective costs from caregivers of child contacts, < 15 years old, who were enrolled in CHIP-TB and consented for the costing study. An initial survey, conducted within one week of trial enrollment, collected data on participant demographics, household socioeconomic status, travel time (in minutes) between the home and health facility, and direct and indirect costs borne by the household. Indirect costs referred to household lost wages or income forgone due to caregivers missing work to be present with the child. In the cost survey, the costing research assistant explained that this could include a missed daily wage for salaried workers or the value of unsold crops or goods for farmers and small business owners. At least two weeks after the anticipated date of TPT completion, a follow-up costing survey captured costs on follow-up care and management of TPT related adverse events. Financial costs associated with debt or selling an asset owing to TPT care were also captured and the proportion of these costs relative to their household income over the same period was calculated. Costing research assistants sought costs borne by the household owing to TPT exclusively. To ensure that only TPT-related costs were captured, costing research assistants explicitly informed survey respondents that they were only interested in expenses incurred specifically for TPT and not for any other TB-related care, including that of the TB client.

As part of the time and motion study, direct observations of TPT and related service delivery times were conducted among a subset of participants who were present at the health facility or their home at the same time as the costing RA. These observations measured the time spent on specific TPT care-seeking activities, such as screening and counselling. Additionally, overall time spent by participants across different TPT care-seeking episodes was recorded.

Health system delivery costs included staffing costs, administrative and implementation costs (such as training, travel, salaries of research staff, external professional services, and other project-related expenses), as well as equipment and printing costs, which covered communication materials, publication costs, and equipment purchased for the study. To capture these costs, we adopted top-down and bottom-up costing methods. We retrospectively analyzed study expenses and conducted key informant interviews of the study team to estimate resource usage and determine whether a resource was owned—either purchased using study funds or borrowed from another project. All upfront project staff time and equipment capital costs were annualized with an estimated useful life of 10 years, discounted at 3% [13]. To assess health system staff costs, we multiplied salaries of health care workers by the proportion of working hours spent on TPT-related programmatic activities. This proportion was assessed via health facility staff surveys, time-and-motion observations, and time stamping of case report forms (CRFs). Section S2.1 of the S1 Text describes how health system delivery costs were calculated in more detail.

To assess the difference in access to care between participating households, we adapted the multidimensional poverty index with minor modifications, through responses from the household cost survey and CRFs, assigning an equal weight (0.33) to health (two indicators), education (2 indicators), and living conditions (6 indicators) [14]. A net index score (out of 1) was utilized to classify the participating households into 5 quintiles, with the most deprived group as “Quintile 1” and the least deprived as “Quintile 5”.

All costs were converted to 2022 USD (1 USD = 51.2 ETB) [15]. A detailed description of cost data collection (S1 Text, section S1), cost data analysis (S1 Text, section S2), and the formulation of the modified multidimensional poverty index (S1 Text, section S.2.2 and Table S3) can be found in the supporting information files.

### Outcomes

Our primary outcome was the difference in the median cost per household initiating at least one child on TPT, comparing the home-based and the facility-based arm. Our secondary outcome of interest was the incremental cost per additional child contact initiated on TPT. Both these outcome measures include costs assessed from both the participant as well as health system perspective. Further, we assessed the financial impact of TPT care on households by calculating the proportion of total financial losses—including direct out-of-pocket costs and indirect losses like debt, asset sales, or missed work—relative to their annual household income. We also performed sub-group analysis based on the travel time between the home and the clinic for households belonging to the five socio-economic quintiles.

### Input data and statistical analysis

Empirical cost and time inputs from trial expense reports, key informants, CRFs, and time-and-motion tools were used to estimate the primary, lower, and upper bound values for input cost categories from a health system perspective. Household cost surveys, administered across all 18 clinics, were used to estimate the primary, lower, and upper bound values of input costs from the perspective of participating households. Mean cluster-level ratios of children on TPT per client with TB, as well as the lowest and highest clinic rates, were used to estimate the primary, lower, and upper bound values of TPT initiation rates.

We conducted a probabilistic sensitivity analysis by performing a Monte Carlo simulation, where we sampled input costs and TPT initiation proportions 1000 times, to obtain 95% uncertainty intervals for the primary and secondary cost endpoints, across both arms, and correspondingly the difference in costs for both points. The primary estimate and ranges for each of the input costs and effects along with their assumed distribution in our model are presented in the supporting information files (Table B in S1 Text). For the primary cost endpoint, we estimated the household and the health system delivery components of the costs across both arms.

We computed the mean and the 95% confidence intervals of the travel time between the home and clinic for each of the five socio-economic quintiles of the costing study population.

This manuscript follows the CHEERS (Consolidated Health Economic Evaluation Reporting Standards) checklist to ensure comprehensive reporting of health economic evaluations. The completed checklist is provided in the supporting information files (S2 Checklist).

All analyses were performed in Microsoft Excel (version 2311) and R (version 4.3.2). The collected data is provided in S1 Data.

### Role of the funding source

The funder of the study, Unitaid, had no role in the study design, data collection, data analysis, data interpretation, or writing of the report.

## Results

**Fig 1:**
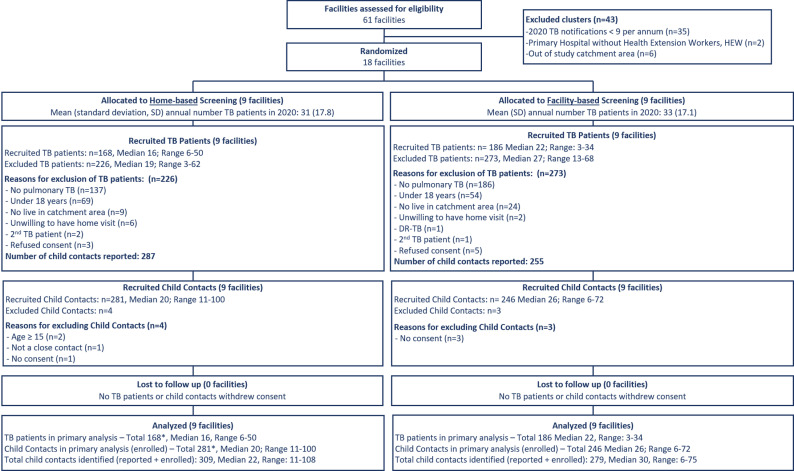
CONSORT Flow Diagram. *One tuberculosis (TB) client with two child contacts was diagnosed with drug-resistant tuberculosis (DR-TB) after TB Preventive Treatment (TPT) completion. The TB client and both contacts therefore remained in all analyses.

125 caregivers from 354 participating households enrolled for the costing study (35% enrolment). Out of the 61 facilities assessed for eligibility, 18 facilities were randomized, nine in each arm. The facilities in the home-based arm had 31 patients on average in 2020, while the facility-based arm had 33. Fig 1 presents the CONSORT flow diagram for all participants in the CHIP-TB trial, while Table 1 summarizes the characteristics of costing study participants across the facility-based and home-based arms.

**Table 1:** Characteristics of costing study participants.

	Home-based	Facility-based
Number of total households analysed in CHIP-TB study	168	186
Number of households enrolled for costing study (% of total HHs)	56 (33%)	69 (37%)
Number of child contacts assessed via costing study	160	158
Mean Number of households enrolled per clinic (range)	6 (0-21)	8 (1-15)
Sex of head of household		
Female (%)	5 (9%)	6 (9%)
Male (%)	51 (91%)	58 (84%)
Not available	0 (0%)	5 (7%)
Sex of survey respondent		
Female (%)	17 (30%)	27 (39%)
Male (%)	39 (70%)	37 (54%)
Not available	0 (0%)	5 (7%)
Age of survey respondent		
18 to 30 years (%)	19 (34%)	24 (35%)
31 to 45 years (%)	25 (45%)	24 (35%)
Above 45 years (%)	12 (21%)	16 (23%)
Not available	0 (0%)	5 (7%)
Socioeconomic assessment		
Households with socioeconomic status assessed (N)	56	64
Normal growth score of child assessed	49 (88%)	47 (73%)
Households without a child death in the past 5 years	54 (96%)	61 (95%)
At least one household member has completed >6 years of education	32 (57%)	44 (69%)
Households with assessed child attending school if eligible^*^	36 (64%)	55 (86%)
Households using solid fuel	0 (0%)	2 (3%)
Households with improved sanitation	9 (16%)	13 (20%)
Households with accessible and safe drinking water	27 (48%)	37 (58%)
Households with electricity	29 (52%)	40 (63%)
Households with adequate floor, roof, and wall materials	27 (48%)	34 (53%)
Households with at least 2 of the following assets: radio, TV, telephone, computer, animal cart, bicycle, motorbike, or refrigerator	43 (77%)	50 (785)

* If child <6 then they will not be deprived on the education scale even if they do not attend school

### Primary outcome

**Fig 2:**
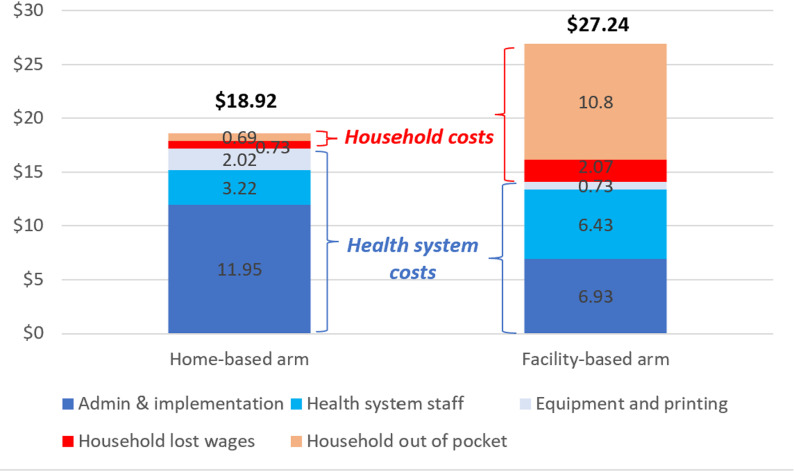
Cost of TPT provision per household. Each colored stack represents a unique cost category. The height of each stack is the contribution of that particular category to the overall cost of TPT provision per household. The bars are stacked from bottom to top in the descending order of their relative contribution to the total cost in the home-based arm. The blue-grey stacks are for cost categories that constitute costs from the perspective of the health system. The red-orange stacks are for cost categories that constitute costs from the perspective of the participant household. Household costs include direct out of pocket costs (“Household out of pocket”) and indirect costs (“Household lost wages”). Health system costs capture human resource costs as “Health system staff” costs, capital costs under “Equipment and printing”, and other overhead and implementation costs under “Admin and implementation”.

There was no notable difference in the health system delivery cost between the two arms, as indicated by the overlapping 95% uncertainty intervals ($17.46 [95% UI: $13.71, $22.02] in the home-based arm vs. $14.28 [95% UI: $11.22, $17.86] in the facility-based arm). In the home-based arm, administration and implementation costs were higher, driven by expenses related to transportation, training, and logistical support. However, staff costs were significantly lower, as less specialized personnel managed TPT delivery. In contrast, the facility-based arm incurred higher staff costs due to the reliance on more experienced healthcare professionals for contact management and TPT administration. The total out of pocket and opportunity costs borne by the household as a result of seeking care for TPT were lesser in the home-based arm ($1.44, 95% UI $0.70, $2.66) compared to the facility based-arm ($12.96, 95% UI $5.27, $22.42). Likewise, the combined cost per household with at least one child initiating TPT was $8.32 lower in the home-based arm ($18.92 95% UI $15.03, $23.68) than the facility-based arm ($27.24, 95% UI $18.59, $39.90). Fig 2 presents the per household cost by cost category. Household costs accounted for nearly half (48%) of costs in the facility-based arm compared to only 8% in the home-based arm. Administration and implementation account for ~ 60% of all costs in the home-based arm. The uncertainty ranges for each cost category are visually represented in section S2.3 of S1 Text.

### Secondary outcome

Table 2 summarizes the total modeled health system and household costs for both the facility-based and home-based arms of our trial, along with the primary and secondary outcomes.

**Table 2:** Primary and secondary cost outcomes.

	Home-based arm (US$)	Facility-based arm (US$)
Median cost and outcomes (95% uncertainty interval)		
Total health system cost (A)	$2909 ($2226, $3854)	$2645 ($1989, $3414)
Total household cost (B)	$244 ($116, $451)	$2414 ($988, $4105)
Total cost (C = A + B)	$3160 ($2455, $4122)	$5064 ($3460, $7047)
Number of eligible households initiating TPT (D)	168 (146, 189)	185 (162, 209)
Total cost per household initiating TPT (E = C/D)	$18.92 ($15.03, $23.68)	$27.24 ($18.59, $36.90)
Number of children initiating TPT (F)	287 (203, 388)	251 (124, 390)
Incremental cost (difference in B)	-$1859 (-$3993, $86)	
Incremental effect (difference in F)	34 (-128, 207)	
Incremental cost per additional child contact on TPT (B/F)	-$10.28 (-$239.24, $343.81)	

The incremental cost effectiveness ratio (ICER), measured as the incremental cost per additional child contact initiated on TPT was -$10.28 (95% UI: -$239.24, $343.81). The wide uncertainty range is due to the similar number of children initiated on TPT in both arms, resulting in a small difference in the denominator, which amplifies the variability in the reported ratio.

**Fig 3:**
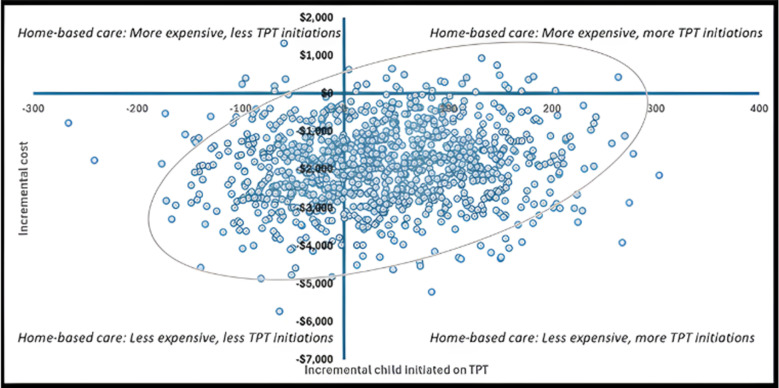
Probabilistic sensitivity analysis plot. The scatter plot has 1000 solid circles or scatters, each representing a single simulation outcome from the probabilistic sensitivity analysis (PSA). The horizontal axis captures the additional children initiated on TPT in the home-based arm compared to the facility-based arm. A filled blue circle on the left side of the y = 0 line signifies a decrease in the number of child contacts initiating TPT when comparing the home-based to the facility-based arm. The vertical axis represents the additional cost spent on the home-based arm compared to the facility-based arm. A filled blue circle below the x = 0 line signifies the home-based arm is cheaper than the facility-based arm. In the bottom right quadrant, the home-based arm is cheaper and more effective than the facility-based arm.

In 61.5% of the simulations home-based care was less costly and more effective than facility-based care (Quadrant IV in Fig 3). Home-based contact management is a cost saving strategy for 96.8% of the simulations. For 35.3% of the simulations, the home-based arm is cheaper and less effective than the facility-based arm.

### Financial dissaving relative to annual household income

In the home-based arm, the median proportion of financial loss due to TPT provision was 0% (IQR: 0%–0.1%) for households. In contrast, in the facility-based arm, the median proportion was higher at 2.7% (IQR: 1.2%–5.2%).

### Travel time by socio economic quintile

Households belonging to the relatively most deprived Quintile 1 (64 minutes, 95% confidence interval, CI 44, 85) on average spent 40 additional minutes to travel to a health facility, when compared to the relatively least deprived Quintile 5 (24 minutes, 95% CI 17, 32). Households in Quintile 2, 3, and 4 spent an average of 48, 43, and 38 minutes respectively.

## Discussion

Our study, conducted prospectively and nested within the CHIP-TB trial, aimed to evaluate the empirical costs of home-based contact management including TPT initiation and follow up relative to a facility-based approach for child contacts <15 years old in Ethiopia, from both the household and the health system perspective. Our findings suggest that home-based contact management by lay CHWs is only modestly more costly to the health system ($17.46 in the home-based arm vs $14.28 in the facility-based arm), while saving substantial costs for households ($1.44 in the home-based arm vs $12.96 in the facility-based arm). The home-based strategy is cheaper and more effective than the facility-based standard of care 61.5% of times. Additionally, we found that households belonging to the relatively most disadvantaged quintile 1 were on average located further away from the clinic compared to the relatively least disadvantaged quintile 5 (64 minutes vs 24 minutes), suggesting that a home-based strategy could reduce the disproportionate economic and financial burden faced by the most disadvantaged households.

In a modelling analysis, Ryckman and others have modelled TPT to be cost-effective for household contacts younger than 5 years ($22 per DALY averted) and contacts aged 5–14 years ($104 per DALY averted) [4]. In study similar to CHIP-TB in Cameroon and Uganda (CONTACT), Mafirakureva and others used trial data and modelled the utility of community-based household tuberculosis contact management using nurses, and demonstrated the incremental cost-effectiveness ratio to be $620 per DALY averted in Cameroon and $970 per DALY averted in Uganda [16]. Our study uniquely leverages CHWs in a task-shared model to support contact management, from TB screening to TPT initiation and completion. As a result, the home-based strategy of TPT provision is cost-saving when including household costs. The significant cost savings from the household perspective for the home-base strategy is largely attributed to the elimination of travel and associated indirect costs, such as opportunity costs owing to missed work, which comprise a substantial portion of the costs in the facility-based model. The existing relationship of the CHWs with the community ensures that home visits could be coordinated in such a way that both the child and the primary caregiver are available for a home visit, effectively leading to dual gains from more child contacts enrolled to care, and caregivers not having to miss work.

In the facility-based arm, household costs accounted for nearly half of the total costs, whereas in the home-based arm, these costs were minimized, representing only 8% of the total costs. On the other hand, the home-based arm incurred higher administrative and implementation costs due to the logistics of deploying CHWs to provide TPT at home. Staffing costs were another major category. The use of CHWs for home-based contact management proved to be more cost-saving than employing programmatic staff in health facilities. The CHWs, who are already monitoring TB clients at the community, could additionally screen and monitor child contacts of the TB client during the same visit to the household. Moreover, the success of the home-based contact management underscores the broader potential for community-based health interventions to address other infectious diseases, including providing TPT to adult household contacts, and chronic conditions, particularly in low-resource settings, and further providing care in an integrated manner [9,17].

While we did see significant differences in travel time between the relatively most and least deprived quintiles, the purpose of this analysis was to highlight the broader benefits of decentralized or household-centered interventions, beyond just economic burden. There exists sufficient literature outlining disproportionately higher costs for TB illness for people with higher levels of poverty [18]. A study by Tadesse and others published in 2013 highlighted low treatment initiation amongst TB clients located far away from health facilities, and thus advocated for more decentralized TB care [19]. A quarter of the households in the facility-based arm reported financial costs associated with TPT were at least 5% of their annual household income. Thus, households facing catastrophic costs owing to TB care may face additional financial burden owing to TPT care at the health facilities. These disparities highlight the importance of considering access barriers when designing health service delivery interventions. Home-based contact management could mitigate these inequities by bringing care directly to households, thus improving access for the most disadvantaged populations. While these findings suggest that the intervention leads to overall societal savings, it is true that costs to the health system may increase. Further research is warranted as to the affordability (i.e., budget impact) of these increases, from a payer perspective, given the number of households that would be eligible for this intervention throughout Ethiopia and the current budget available.

One of the primary strengths of our study is the pragmatic design of the main trial and our parallel, prospective assessment of empiric costs assessed from the partial societal perspective, which enhances the generalizability of our findings. Secondly, the comprehensive data collection, including economic costs, time data, and equity measures, allows for a thorough assessment of the intervention’s impact from multiple perspectives. Lastly, the presentation of results from both the household and the health system perspective will allow policymakers to assess how modest investments in the health system could reap significant economic gains for households.

Our study should be interpreted in light of certain limitations that may reduce the generalizability of our findings. First, while the use of CHWs appears cost-effective, our evaluation may not fully capture the implementation challenges of home-based contact management. In particular, healthcare worker time—rather than financial resources—may become the primary constraint, adding to the existing workload of an already overburdened community health workforce. A post-trial qualitative assessment will further explore this limitation and will be reported separately. Secondly, in the home-based arm, two study clinics in the main trial did not participate in our costing study. Although the exclusion of these two clinics may introduce some bias into our findings, we do not have data from these sites to assess whether their inclusion would have significantly impacted the results. Thirdly, our study was conducted in a rural setting, where home-based care may have been more acceptable due to greater distances to health facilities. In urban areas, where healthcare facilities are more accessible, individuals may have had a stronger preference for facility-based care. Finally, our sample size was based on logistical feasibility, given the limited time available for research assistants to conduct cost surveys of clients. While we hoped for a larger sample size, there was not a specific target - and we note that our existing sample size was sufficient to provide precision of + /-50% around all of our primary estimates, a precision that we believe is sufficient to support our key conclusions.

Future studies could further explore the value of a hybrid TPT service delivery model that combines both home and facility-based approaches. In a recent trial in Uganda, when people living with HIV were given a choice between clinic-based directly observed therapy (DOT) and home-based self-administered therapy (SAT), many participants chose clinic-based DOT [20]. Whereas in our study, conducted in a predominantly rural setting, more children were initiated on TPT in the home-based arm. This suggests that TPT provision might benefit from an optimized combination of both strategies. A hybrid model that considers geographical accessibility to health facilities, respects household preferences, and minimizes strain on health system resources could be a viable option. Further, optimized home-visit operations will be key in further improving the cost-savings nature of the home-based intervention [21].

## Conclusion

In conclusion, home-based contact management offers a cost-saving alternative for households and maintains similar completion rates to facility-based care. This approach can enhance access to TPT, particularly for socio-economically disadvantaged populations, and contribute to closing the equity gap in TB care. However, careful consideration of the associated health system costs and ethical implications is essential. Future studies should expand on these findings to inform budget implications linked to the scale-up of home-based TB interventions globally.

## Supporting information

S1 Checklist Additional information regarding the ethical, cultural, and scientific considerations specific to inclusivity in global research. (DOCX)

S1 Text This document provides details on cost data collection and classification methods for both health system and household costs. Fig A in S1 Text. **Breakdown of TPT Provision Costs by Arm and Cost Category.** The chart illustrates the per-household cost of TPT provision, comparing home-based (blue) and facility-based (orange) approaches. Costs are divided into health system costs (including administration, implementation, staff, and equipment) and household costs (including lost wages and out-of-pocket expenses). The bars represent the average costs for each category, with 95% uncertainty intervals indicated by the error bars. Table A in S1 Text. **Tagging of health system costs and trial costs.** This table classifies the proportion of each cost input allocated to programmatic versus research activities, the proportion attributed to the implementation versus control arm, and the proportion attributed to the specific clinics included in the analysis. Table B in S1 Text. **Point estimate, lower bound, and upper bound of per test cost components in 2022 USD.** This table presents the point estimate, lower bound, and upper bound for each cost category in both the intervention and control arms. These values were used as inputs for the Probabilistic Sensitivity Analysis. Table C in S1 Text. **Scoring on the Modified Multidimensional Poverty Index.** This table details how indicators across the three dimensions of poverty, i.e., health, education, and standard of living, contributed to the calculation of the modified multidimensional poverty index score for each household.
(DOCX)

S2 Checklist This document outlines the reporting standards followed in the economic evaluation, based on the Consolidated Health Economic Evaluation Reporting Standards (CHEERS) checklist. (DOCX)

S1 Data This excel spreadsheet compiles the input household and health system cost data. (XLSX)
