# Supplementary material for: Cost and cost-effectiveness of pediatric home-based versus facility-based TB Preventive Treatment in Ethiopia (CHIP-TB)
Source: PLOS Glob Public Health. 2025 Apr 30;5(4):e0004466. doi: 10.1371/journal.pgph.0004466 (PMC12043179; doi:10.1371/journal.pgph.0004466)
Supplement: S1 Text — Fig A in S1 Text. Breakdown of TPT Provision Costs by Arm and Cost Category. The chart illustrates the per-household cost of TPT provision, comparing home-based (blue) and facility-based (orange) approaches. Costs are divided into health system costs (including administration, implementation, staff, and equipment) and household costs (including lost wages and out-of-pocket expenses). The bars represent the average costs for each category, with 95% uncertainty intervals indicated by the error bars. Table A in S1 Text. Tagging of health system costs and trial costs. This table classifies the proportion of each cost input allocated to programmatic versus research activities, the proportion attributed to the implementation versus control arm, and the proportion attributed to the specific clinics included in the analysis. Table B in S1 Text. Point estimate, lower bound, and upper bound of per test cost components in 2022 USD. This table presents the point estimate, lower bound, and upper bound for each cost category in both the intervention and control arms. These values were used as inputs for the Probabilistic Sensitivity Analysis. Table C in S1 Text. Scoring on the Modified Multidimensional Poverty Index. This table details how indicators across the three dimensions of poverty, i.e., health, education, and standard of living, contributed to the calculation of the modified multidimensional poverty index score for each household. (DOCX) [file pgph.0004466.s002.docx]

**APPENDIX**

# **S1 Cost data collection**

## ***S1.1 Health system costs***

A combination of time-and-motion forms, staff time sheets, Government salary records, time-stamping of case report forms (CRFs), and trial expense reports were analysed to assess the health system component of the cost per child initiated on TPT.

S1.1.1 Time-and-motion (TAM) forms

Participant episodes at the clinic and household were recorded by costing research assistants using time-and-motion (TAM) forms. The forms were prepared both on paper and REDCAP. If research assistants were not able to capture the data directly on the REDCAP Form

S.1.1.1.1 TAM at the health center

The following details were first captured:

1. Time of arrival at health center (retrospective entry after checking with participant)
2. Date of arrival
3. Health center number
4. Pin of the child
5. Confirmation of consent by observed caregiver, child, and health care worker
6. Type of visit (whether the first visit, a follow up visit, a visit due to an adverse event, or a visit du to another reason)

Next, for each episode or activity, we captured the health care worker involved (the role/title of the worker), the activity ID, the start time, and the end time. A key for acceptable activity IDs was provided to the research assistants.

S.1.1.1.2 TAM during home visits

The following details were first captured:

1. Date of visit
2. Initials of health extension worker
3. Health center number (home is in the catchment area of this health center)
4. Name of nearest health post
5. Pin of the child

Next, for each episode or activity, we captured the activity ID, the start time, and the end time. A key for acceptable activity IDs was provided to the research assistants.

S1.1.2 Staff time survey

The costing research assistants met with the clinic/site administrator to prepare a list of all staff, including the volunteer staff at that clinic/site who were involved with the CHIP TB study, even if for a part of their time. The job title and approximate start date of employment for each staff member was recorded.

From the list prepared, the costing research assistants would, depending on the convenience and availability of the staff, survey them on how they spend their work week. Staff members were eligible to be surveyed multiple times. The survey was aimed at estimating the proportion of a staff member’s time spent on CHIP-TB specific activities versus routine programmatic duties throughout the study period. The following details were captured:

1. Date
2. Job title
3. Employment status
4. Work experience (overall and at clinic)
5. Expected number of working hours
6. Number of working hours worked during the prior week
7. Activities performed during the prior week

S1.1.3 Time stamped forms

## We recorded the start and end times for each case report form (CRF) and informed consent document. This enabled us to estimate the time spent entering data specifically for research purposes. By doing so, we could subtract this research-related time from the total time, leaving us with an estimate of the time spent on programmatic activities.

## S1.1.4 Government salary records

Salary bands of healthcare workers, which varied by seniority and years of experience were accessed by the study team to estimate what proportion of their salary was used exclusively for CHIP TB Research, and what proportion could be appropriated to other programmatic activities.

S1.1.5 Trial expense reports

Trial expenses for CHIP TB in Ethiopia were accessed and reported as costs for:

1. Communication material and publication
2. External Professional Services
3. Project Staff
4. Travel and Training
5. Equipment other than health related equipment
6. Other project expenses
7. Indirect costs (12% of all other costs)

## ***S1.2 Household costs***

The primary sources of cost data collection at the household were the initial and follow up household cost surveys. A few costs (time) were triangulated with the time and motion forms.

S1.2.1 Household cost surveys

This survey was administered to participants at both the intervention and control clinics.

S1.2.1.1 Initial household cost survey

The survey captured participant demographics, household characteristics, and travel time between the household and health center. Direct household out of pocket costs such as costs for food, transport, childcare, other costs (borne by everyone from the household who accompanies the child, including the child, for the TPT specific visit) are captured only if any additional expenses were made owing to TPT only, i.e. they would not have made these expenses if the child in their household was not initiated to TPT. Indirect costs, because of missed work as a result of TPT delivery, were also captured. A socio-economic assessment was conducted at the end of the survey. The assessment captured the educational attainment of household members, dwelling characteristics, and assets owned by the household. Since this survey was conducted within a week of enrolment, exact cost values were queried.

S1.2.1.2 Follow-up household cost survey

The follow up household cost survey focused on the direct out-of-pocket costs and indirect costs due to missed work borne during the duration of the TPT delivery. The survey captured the nature and severity of adverse events, and costs owing specifically because of the respective adverse events. Catastrophic costs linked to TPT, such as loss of job or productivity at work, sale of property, and borrowing of loans, all because of caring for child on TPT, were also recorded. Household income was also captured. Since this survey was conducted within a month of participant endpoint, approximate costs over the duration from the first survey till the endpoint were captured.

S.1.2.2 Time and motion

Though time-and-motion tools primarily assessed time (and as a result costs) borne by the health system, these could also be used to triangulate participant time spent on TPT service delivery.

Both the clinic and home based forms collect data on the amount of time participants spend interacting with healthcare workers, including the specific nature of the activity (to assess if the activity is TPT related). Additionally, the clinic based form captures the travel time from the health center to the household, just as it is recorded in the household cost surveys.

# **S2 Cost data analysis**

Through various data collection methods listed in the prior section, we built up a deterministic cost of TPT initiation per household.

## ***S2.1 Health system component of per household cost***

S1.1.1 Finalization of cost categories

While reporting the costs, we clubbed certain cost categories to eventually report costs as:

1. Health system staffing costs
2. Admin and implementation costs: Includes expenses on training, travel, salaries of research staff, external professional services, and other project expenses.
3. Equipment and printing costs: Includes communication materials, publication costs, equipment purchased for the study

S2.1.2 Classifying health system costs as research vs programmatic

Discussions with the study team (PI, site PI, study coordinators) helped us come up with a first pass of what might be purely research costs, what might be programmatic, etc. Even if a cost was a purely research cost, we considered a proportion of these costs as programmatic (as these particular activities lead to a programmatic “effect”). Next, for most cost categories, we divided these costs by an impact factor, to attribute what proportion of the cost could be attributed to clinics being analyzed in our study. We had point estimate, upper, and secondary bounds for these inputs. For example: if there was a particular cost for a consultant for the qualitative study, inputs from the study team suggested we attributed 25% of these costs as programmatic costs (And 75% as research). Then, if we assume the impact of the study will reach all other clinics in the province, we divide this proportion by 50 (since there are approximately 50x health centers spread across a typical province; base case); if the impact is only to a small province, then we divide proportion by 10 (upper bound), and if the impact is national then we divide this proportion by 200 (lower bound). For certain upfront costs, such as equipment, the impact factor was the estimated life years (ELY) of the equipment, and the costs were annualized using a discount rate of 3%. Training costs and salaries of research assistant were also annualized using the same approach. The ELY was 10 years for the point estimate, 5 years for the upper bound value, and 20 years for the lower bound value.

S.2.1.3 Classifying health system costs as intervention vs control

Next, we had to determine whether the costs were tagged under the home-based intervention arm or the facility-based standard of care or control arm.

For trial expense reports: We leveraged the time and motion data, as well as data from time stamped forms to see how many minutes per household were spent on programmatic activities and programmatic data collection. From the time and motion forms, of all the time spent on programmatic activities, 60% was in the intervention arm while 40% was in the control arm. On analyzing the time stamped CRFs, we were able to attribute 63% of programmatic data entry to the intervention arm, and 37% to the control arm. Thus, if a reported cost indicated field work, we first estimated the programmatic component of that cost and then attributed 60% to the intervention arm and 40% to the control arm. If the cost was an “overall study cost” with no field related component, we split the cost equally (50-50) across both the arms. We attributed all the costs to the intervention arm if we were analyzing the costs of equipment (such as file cabinets) that were procured solely for the intervention arm.

The table below describes what proportion of a cost was categorized as research vs. programmatic, what proportion of the costs was categorized as intervention vs control, and what was the impact factor of each health system cost item. The point estimate (or the lower and upper bound) for each cost item across each arm was calculated as: [(Reported cost)*(Proportion of programmatic components)*(Proportion of intervention/control)]/[Impact Factor for point estimate or upper bound or lower bound). The calculated values belonging to similar cost categories were clubbed together and presented (as described in section S.2.1.1).

Table A. Tagging of health system costs trial costs.

| Cost item | Cost (USD) | % Programmatic | % Intervention arm | Impact factor: point estimate (high, low) |
| --- | --- | --- | --- | --- |
| Contract staff (1 Research Assistant) | 7,149 | 25% | 50% | 50(10, 200) |
| Research Assistant (3 Costing RAs) | 4,477 | 0% | 50% | 10(5, 20) |
| Consultant payment - Qualitative Study Team(2 Interviewers, 2 transcribers & 2 translators) | 21,824 | 25% | 60% | 50(10, 200) |
| Communication/Internet, telephone | 1,880 | 25% | 50% | 50(10, 200) |
| Office Rent, utility, office supplies, etc | 26,470 | 25% | 50% | 50(10, 200) |
| Research Assistant ( Nine) | 125,770 | 25% | 60% | 10(5, 20) |
| Finance Officer | 9,148 | 25% | 60% | 50(10, 200) |
| Country PI | 68,487 | 10% | 60% | 50(10, 200) |
| Project/Study Coordinator | 45,538 | 25% | 60% | 50(10, 200) |
| Central Office Co-PI | 26,881 | 10% | 50% | 50(10, 200) |
| Central Office grant administration | 19,725 | 0% | 50% | 50(10, 200) |
| Travel & Training | 1,612 | 25% | 60% | 50(10, 200) |
| Central office - international TA | 515 | 25% | 50% | 50(10, 200) |
| Community Advisory board meeting | 9,267 | 25% | 50% | 50(10, 200) |
| TB Focal & HEW Training | 16,941 | 100% | 60% | 10(5, 20) |
| Distribution of study material | 2,421 | 25% | 60% | 50(10, 200) |
| Costing study training | 1,954 | 0% | 50% | 10(5, 20) |
| Supportive supervision/Site visit | 25,927 | 25% | 60% | 10(5, 20) |
| Study Related training | 30,609 | 25% | 60% | 50(10, 200) |
| Qualitative Interview data Collection | 2,191 | 25% | 60% | 50(10, 200) |
| Equipment other than Health related | 1,801 | 25% | 50% | 10(5, 20) |
| Tablets for research assistants (9) | 2,594 | 25% | 60% | 10(5, 20) |
| Communication/staff mobile card | 3,023 | 25% | 60% | 10(5, 20) |
| 1 laptop for project coordinator | 1,199 | 25% | 50% | 10(5, 20) |
| 1 Table/desk for Project coordinator | 307 | 0% | 50% | 10(5, 20) |
| Hand Sanitiser (study team use) | 866 | 0% | 50% | 10(5, 20) |
| Face Mask (study team use) | 864 | 0% | 50% | 10(5, 20) |
| 18 Metallic File Cabinet for Health Center | 4,402 | 5% | 50% | 10(5, 20) |
| 45 File Cabinet for Health Post | 6,101 | 5% | 100% | 10(5, 20) |
| 45 Small digital weight scale for health posts (for HEWs use) | 2,300 | 100% | 100% | 10(5, 20) |
| Printing of study materials (CRFs, ICFs) | 10,174 | 5% | 60% | NA (effective cost varied by 50% for lower and upper bound values) |

For staff salaries: Through inputs from the study team, time and motion surveys, staff time surveys, and Government salary records, we were able to attribute a component of a healthcare worker’s salary to the programmatic time they spend on delivering TPT. The salary proportions of lab technicians and OPD workers were split equally across both arms. The salary proportions of health extension workers were attributed solely to the intervention arm and the salary proportions of TB focal persons were attributed solely to the control arm.

In calculating the cost per household within the study catchment areas, we included all households, not just those with at least one eligible child contact, to ensure a comprehensive and accurate assessment.

## ***S2.2 Participant component of cost per household***

S2.1.1 Finalization of cost categories

All direct out of pocket costs were clubbed together as one category called “Household out of pocket costs”. While the indirect costs were categorized as “Household lost wages”.

S2.1.2 Total financial expenditure as a proportion of annual income

To calculate the total financial expenditure, we added the direct and indirect costs as reported above, including lost wages due to loss of employment. Further, if the household sold property or borrowed money to pay for TPT care, we considered 10% of the cost value to be financial costs. We took a proportion of these costs over the total annual household income, and presented the median and the interquartile range.

## ***S2.3 Cost inputs for Probabilistic Sensitivity Analysis***

Table B. Point estimate, lower bound, and upper bound of per test cost components in 2022 USD.

|  | Intervention ($) | | | Control ($) | | |
| --- | --- | --- | --- | --- | --- | --- |
|  | PE | Lower | Upper | PE | Lower | Upper |
| Communication material and Publication | 0.78 | 0.32 | 1.46 | 0.44 | 0.18 | 0.83 |
| External Professional Service | 0.21 | 0.04 | 1.33 | 0.13 | 0.03 | 0.84 |
| Other Project Expense | 0.18 | 0.04 | 1.13 | 0.15 | 0.03 | 0.97 |
| Project Staff | 6.32 | 2.83 | 17.45 | 3.63 | 1.62 | 10.08 |
| Travel & Training | 4.54 | 2.07 | 11.91 | 2.61 | 1.19 | 6.91 |
| Equipment (non-health) | 1.17 | 0.56 | 2.73 | 0.27 | 0.13 | 0.62 |
| HS Staff costs | 3.10 | 1.57 | 6.18 | 6.27 | 2.94 | 11.88 |
| Household out of pocket | 0.61 | - | 7.70 | 9.94 | - | 31.76 |
| Lost wages | 0.59 | - | 11.55 | 1.76 | - | 17.32 |
| Initiation rates (child initiated per client with TB) | 1.66 | 1 | 3.00 | 1.34 | 0.24 | 2.48 |

We conducted a probabilistic sensitivity analysis by sampling each cost and effectiveness value from a corresponding distribution 1,000 times; 95% uncertainty ranges were defined as the 2.5^th^ and 97.5^th^ percentiles of results across these simulations. For each parameter, we constructed a beta distribution around the empirically observed point estimate, with the weighted mean serving as the mode of the distribution and range based on the minimum and maximum observed values for each parameter in the trial. We assumed alpha = 4 for all parameters (which corresponds to a 95% confidence interval covering 63% of the width of the full distribution, when the beta distribution was symmetric)

## ***S2.4 Alternate representation of cost components of cost per household initiating TPT***


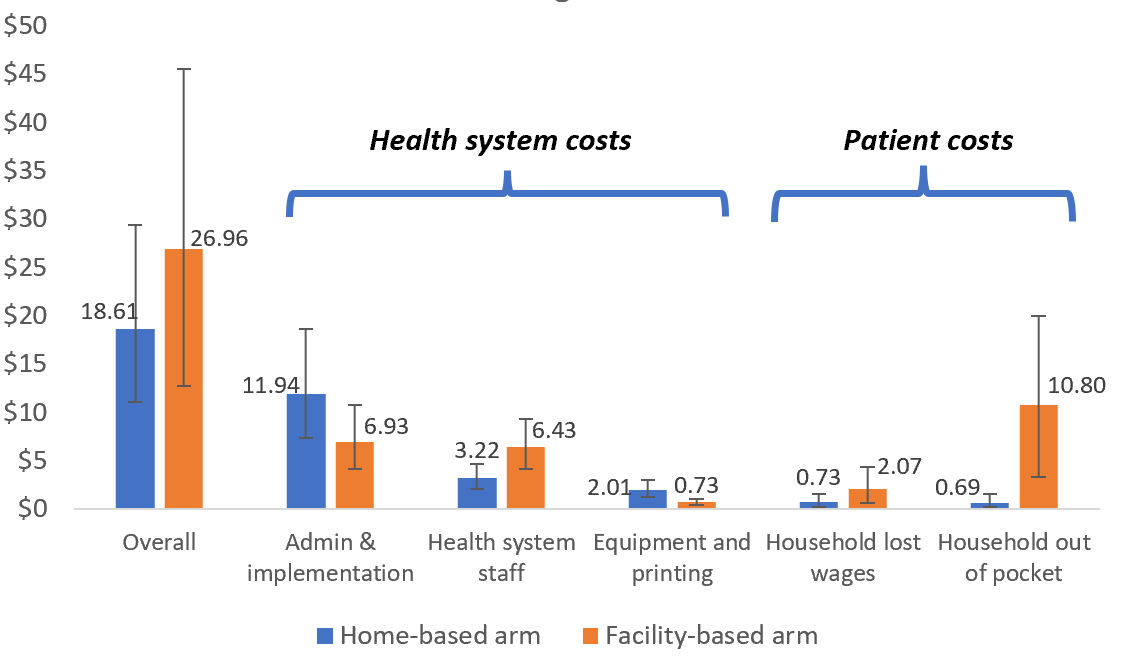


Fig A. **Breakdown of TPT Provision Costs by Arm and Cost Category.** The chart illustrates the per-household cost of TPT provision, comparing home-based (blue) and facility-based (orange) approaches. Costs are divided into health system costs (including administration, implementation, staff, and equipment) and household costs (including lost wages and out-of-pocket expenses). The bars represent the average costs for each category, with 95% uncertainty intervals indicated by the error bars.

# **S3 Time data collection and analysis**

## ***S3.2 Estimating participant travel time***

From the participant cost surveys, we captured the average travel time between the home and clinic, per household.

## ***S3.2 Modified Multidimensional Poverty Index***

Using an adapted approach from the Multidimensional Poverty Index, we created a Modified Multidimensional poverty Index (MMPI) using the participant data collected in our study. Like the MPI, we attributed a third of the weight of the net score to health, a third to educational attainment at the household, and a third to the socio economic status of the household. The table below lists how the index was generated for each household.

Table C. Scoring on the Modified Multidimensional Poverty Index

| Dimension of Poverty | Indicator | Deprived if living in the household where… | Score |
| --- | --- | --- | --- |
| Health | Growth assessment | Child is not normal on the growth assessment chart OR unknown/other assessment. | 0 if not normal, 1/6 is normal, and 1/12 if assessment unknown/other. |
|  | Child mortality | If any child has died in the household in the past 5 years. | 1/6 if not deaths. Score = 0 if at least 1 child has died. |
| Education | Years of schooling | If no adult household member has completed at least 6 years of any form of schooling. | 0 if all adult household members are deprived. Score = 1/6 if at least one adult household member is not deprived |
|  | School attendance | Children 6 or over have not attended school over the past year. | 0 if at least one eligible school age child did not attend school (deprived). Else the score is 1/6 |
| Standard of living | Cooking fuel | The household cooks with dung, wood, charcoal or coal. | 0 if using at least one of the listed fuels. Score = 1/18 if using a clean fuel source. |
|  | Sanitation | The household’s sanitation facility is not improved (according to SDG guidelines) or it is improved but shared with other households | 0 if with unimproved/shared sanitation. 1/18 if improved and individual sanitation. |
|  | Drinking water | The household does not have access to improved drinking water (according to SDG guidelines) or improved drinking water is at least a 30-minute walk from home, round trip. | 0 if unimproved drinking water or source located far away. Score =1/18 if improved and accessible source. |
|  | Electricity | The household has no electricity | 0 if no electricity, 1/18 otherwise. |
|  | Housing | At least one of the three housing materials for roof, walls and floor are inadequate: the floor is of natural materials and/or the roof and/or walls are of natural or rudimentary materials. | 0 if inadequate housing materials, 1/18 otherwise |
|  | Assets | The household does not own more than one of these assets: radio, television, telephone, computer, animal cart, bicycle, motorbike or refrigerator, and does not own a car or truck | 0 if 1 or lesser assets owned. 1/18 otherwise. |

Each household was assigned a score based on the methodology in the table listed above. Households were then split into 5 quintiles based on their MMPI score, with Quintile 1 (Q1) referring to the relatively most disadvantaged quintile and Q5 referring to the relatively least disadvantaged quintile.

## ***S3.3 Average travel time by quintile***

For the households classified in each respective quintile, we computed the average travel time between the home and the health facility.

# **S4 Code**

The code for the analysis can be accessed here: <https://github.com/akash210593/CHIPTBecon>
